# Prompt Engineering and Model Selection for LLM-Based Nutritional Estimation from Food Images: A Multi-Dataset Investigation

**DOI:** 10.3390/nu18122017

**Published:** 2026-06-21

**Authors:** Shinichi Nakagawa, Akira Yamamoto

**Affiliations:** 1 Research Institute of Info-Communication Medicine (RinCOM), Tokyo 184-0004, Japan; 2 Faculty of Health Data Science, Juntendo University, Tokyo 113-8421, Japan; akirayamamotokuhp@gmail.com

**Keywords:** large language model, food image, nutritional estimation, prompt engineering, dietary assessment, Claude, SNAPMe, JBFD, NutriImage

## Abstract

**Background/Objectives:** Accurate estimation of nutritional content from food images has important applications in dietary assessment and public health surveillance. While large language models (LLMs) have shown promise for this task, the effects of prompt design and model selection on estimation accuracy remain poorly characterized. **Methods:** We evaluated three Claude models (Haiku 4.5, Sonnet 4.6, Opus 4.6) for visual estimation of five mandatory nutritional components (energy, protein, fat, carbohydrate, and salt equivalent) across three datasets: NutriImage (691 Japanese meal photographs with dietitian-validated ground truth, after OCR-mask quality filtering), SNAPMe (1463 US meal photographs from a publicly available benchmark), and the Japan Branded Food Database (JBFD; 989–1000 packaged food product images). We systematically compared a default prompt and a visual estimation prompt explicitly instructing the model not to read any text or numbers visible in the image. **Results:** The visual estimation prompt substantially improved accuracy when paired with a sufficiently capable model (energy R^2^: 0.23 for Haiku to 0.60 for Sonnet, JBFD). Sonnet and Opus substantially outperformed Haiku across all datasets, while differences between Sonnet and Opus were small (MedAPE difference 1–3 percentage points). Packaged food images (JBFD) yielded higher R^2^ than meal photographs. Salt equivalent showed consistently poor accuracy (MedAPE 34–64%). On SNAPMe, Sonnet achieved lower energy MAE (116.9 vs. 123.0 kcal, −4.9%) and lower MAE for protein (5.9 vs. 7.9 g, −25.7%) and fat (6.6 vs. 8.7 g, −24.5%) compared with a recent ChatGPT-5 study. **Conclusions:** Claude Sonnet offers the best cost-performance balance for LLM-based nutritional estimation. Prompt design substantially affects accuracy, but only when paired with a sufficiently capable model; model visual recognition capability appears to be a key determinant of performance. These findings highlight the inherent difficulty of this task and provide practical guidance for dietary assessment system development.

## 1. Introduction

Accurate dietary assessment is fundamental to nutritional epidemiology, clinical nutrition management, and public health surveillance. Traditional methods such as 24 h dietary recalls and food frequency questionnaires are subject to recall bias, participant burden, and systematic underreporting [1,2]. Image-based dietary assessment offers an alternative approach by leveraging food photographs to reduce reliance on self-report, and has attracted increasing attention with the proliferation of smartphone technology [3,4].

Image-based dietary assessment systems such as FoodLog, developed by Aizawa and colleagues, have demonstrated that smartphone-based food recording tools using image recognition substantially reduce user burden compared to text-based methods [5]. These systems rely on matching food photographs to curated databases of known nutritional values rather than directly estimating nutrition from visual appearance, a fundamentally different approach from the LLM-based estimation evaluated in the present study.

Recent advances in multimodal large language models (LLMs) capable of processing both images and text have opened new possibilities for automated nutritional estimation from food photographs. Rodriguez-Jimenez et al. assessed ChatGPT-5 (as described by the authors) across four input scenarios using 195 dishes from the SNAPMe dataset, reporting mean absolute errors of 123.0 kcal for energy and 7.9 g for protein under image-only conditions [6]. Fridolfsson et al. compared ChatGPT-4o, Claude 3.5 Sonnet, and Gemini 1.5 Pro using 52 standardized food photographs, finding that ChatGPT and Claude achieved similar accuracy while Gemini showed substantially higher errors [7]. However, these studies share important limitations: small sample sizes (52–195 items), manual evaluation through web interfaces, and limited investigation of how prompt design affects estimation accuracy.

Prompt engineering has been shown to substantially affect LLM performance across a wide range of tasks [8]. Whether to instruct the model to read visible text on food packages versus to estimate from visual characteristics alone has not been systematically investigated. Furthermore, the cost-performance tradeoff across model tiers remains uncharacterized for this task.

**Figure 1:**
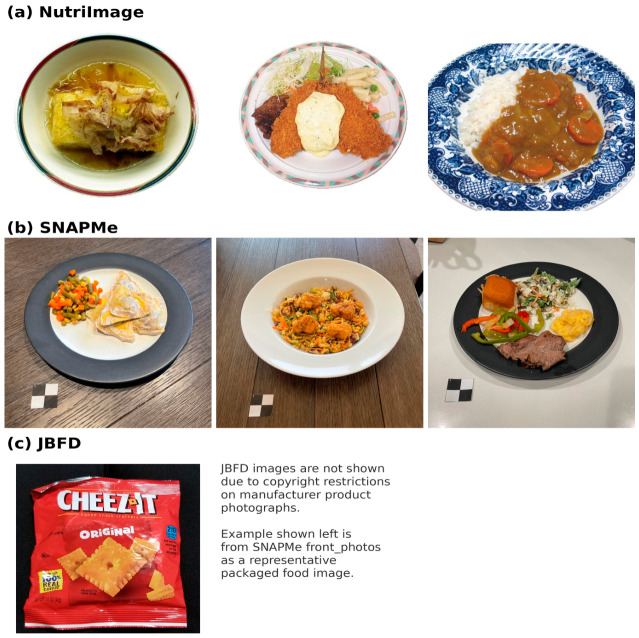
Representative food images from the three datasets (NutriImage: pre-masking originals). (**a**) NutriImage: pre-masking originals showing visible Japanese dish-name and size codes subsequently masked for analysis. (**b**) SNAPMe: US meal photographs in real-life settings with sizing marker. (**c**) Packaged food image example from SNAPMe front_photos; JBFD images not shown due to copyright restrictions.

In this study, we evaluated the effect of prompt design and model selection on LLM-based nutritional estimation across three diverse datasets. We compared two prompt conditions and three model tiers to provide actionable guidance for researchers implementing LLM-based dietary assessment systems. Representative images from each dataset are shown in Figure 1.

## 2. Materials and Methods

### 2.1. Datasets

Three datasets were used in this study (Figure 1).

#### 2.1.1. NutriImage

The NutriImage dataset was developed by the authors as part of the Kenko Eiyou Information System (health-info.jp), an image-based nutritional calculation system [9]. The database contains 886 food items with corresponding meal photographs and nutritional values calculated by registered dietitians. Of 881 items with complete nutritional data, 903 images were available in the masked image directory (including items added after the initial completeness filter). Initial inspection of representative images (Figure 1) revealed that NutriImage photographs contain visible Japanese text labels (dish name and size codes). Text regions were masked using OCR-based detection (pytesseract (v0.3.10)), Japanese language model) with white-fill inpainting of detected bounding boxes. A subsequent quality audit using a Claude Haiku-based vision classifier identified 191 of 903 masked images (21.2%) as retaining visible text; these were excluded, leaving 712 clean images. Of these, 691 had matching nutritional ground truth records and were used for the final analysis.

#### 2.1.2. SNAPMe

The Surveying Nutrient Assessment with Photographs of Meals (SNAPMe) database is a publicly available benchmark dataset [10]. We used the 1478 before photographs of non-packaged foods, with nutritional ground truth from ASA24 food records aggregated per photograph. All items with available images were included (N = 1463 after excluding 15 items with API errors). Images larger than 3 MB were resized prior to API submission to comply with the 5 MB base64 limit.

#### 2.1.3. Japan Branded Food Database (JBFD)

The JBFD was constructed by web-crawling product pages of approximately 30 Japanese food manufacturers [11]. For prompt development, 300 items from the Co-op/JCCU subset were used as a preliminary experiment. For the main model comparison, 1000 items were randomly sampled (random seed = 123) from all 8374 items with available images and complete nutritional data; 989–1000 items yielded valid API responses depending on the model.

### 2.2. Nutritional Estimation Models

Three Claude models were evaluated: Haiku 4.5 (claude-haiku-4-5-20251001), Sonnet 4.6 (claude-sonnet-4-6), and Opus 4.6 (claude-opus-4-6; Anthropic, San Francisco, CA, USA). API costs are approximately $0.80, $3.00, and $15.00 per 1M input tokens, respectively. All estimations were performed via the Anthropic Messages API (v2023-06-01; Anthropic, San Francisco, CA, USA; https://docs.anthropic.com, accessed on 20 May 2026) with images encoded as JPEG base64. Target nutritional components were energy (kcal), protein (g), fat (g), carbohydrate (g), and salt equivalent (g). Sonnet was selected as the primary model for the main experiments based on preliminary cost-performance analysis.

### 2.3. Prompt Conditions

#### 2.3.1. Default Prompt

The model was instructed to estimate nutritional content from the food image without explicit guidance regarding text reading (full text in Appendix A.1).

#### 2.3.2. Visual Estimation Prompt

The model was explicitly instructed to estimate based solely on visual characteristics and not to read any text, labels, or numbers visible in the image. The model was also asked to provide a one-sentence explanation of its reasoning. Full text available at https://github.com/shnkgw-rincom/food-image-nutrition-llm (accessed on 20 May 2026).

A preliminary experiment (N = 300, JBFD Co-op subset) revealed that under a label-reading prompt, Claude Sonnet returned null for 298 of 300 items, confirming that JBFD images predominantly show front-of-package designs without visible nutrition facts panels. The visual estimation prompt was developed in response to these findings. A Chain-of-Thought (CoT) prompt was also evaluated; results are reported in Appendix A.4.

### 2.4. Statistical Analysis

Estimation accuracy was evaluated using R^2^, mean absolute error (MAE), and median absolute percentage error (MedAPE). R^2^ was computed as the coefficient of determination from ordinary least squares linear regression of predicted values on observed values (with intercept), using scikit-learn LinearRegression. MedAPE was selected as the primary percentage-error metric due to its robustness to near-zero true values, which inflate MAPE particularly for salt equivalent. All nutrient estimates and reference values are expressed per 100 g edible portion. The proportion of null (non-numeric) API responses was recorded for each model–prompt combination. All analyses used Python (v3.12; Python Software Foundation, https://www.python.org, accessed on 20 May 2026) with NumPy (v1.26), scikit-learn (v1.4), and pytesseract (v0.3.10).

## 3. Results

### 3.1. Effect of Prompt Design and Model Selection

Table A1 (Appendix A.2) presents R^2^ values from the preliminary experiment comparing prompt conditions and models on the JBFD Co-op subset (N = 300). The visual estimation prompt substantially improved performance for Sonnet relative to Haiku default (energy R^2^: 0.235 for Haiku default to 0.844 for Sonnet visual). Note that Sonnet’s default could not be evaluated at scale as it returned null for most items (see Section 2.3); therefore, this comparison reflects the combined effect of prompt change and model upgrade. Haiku showed no improvement with the visual prompt (energy R^2^: 0.235 to 0.170), indicating that model visual recognition capability, not prompt design alone, determines performance.

A notable behavioral difference emerged: Haiku returned numerical estimates for all items regardless of label visibility, producing a modal energy estimate of 280 kcal for 17.7% of items, consistent with confabulation. Sonnet correctly returned null when nutritional labels were not visible, demonstrating superior epistemic calibration.

### 3.2. Model Comparison Across Datasets

Table 1 presents R^2^ and MAE, and Table 2 presents MedAPE, for all five nutritional components across three datasets and three models using the visual estimation prompt.

For JBFD packaged food images, Sonnet achieved the highest R^2^ for energy (0.603), fat (0.625), and carbohydrate (0.699), with Opus performing comparably (energy 0.586, carbohydrate 0.702). For meal photographs (NutriImage and SNAPMe), Opus showed slightly higher R^2^ for energy (NutriImage: 0.506 vs. 0.480; SNAPMe: 0.530 vs. 0.470). However, MedAPE differences between Sonnet and Opus were consistently small (1–3 percentage points) across all conditions, with no clinically meaningful advantage for the more expensive model.

Salt equivalent showed consistently poor accuracy across all models and datasets (MedAPE 34–64%), reflecting the fundamental difficulty of estimating sodium content from visual information alone.

### 3.3. Comparison with Prior Work on SNAPMe

Table 3 compares Sonnet results on SNAPMe with those of Rodriguez-Jimenez et al. [6]. Our approach achieved lower energy MAE (116.9 vs. 123.0 kcal, −4.9%) and lower MAE for protein (5.9 vs. 7.9 g, −25.7%) and fat (6.6 vs. 8.7 g, −24.5%), using a fully automated pipeline on a larger sample (N = 1463 vs. N = 195 manual). Carbohydrate MAE was higher (14.4 vs. 11.7 g, +23.1%), likely reflecting the diversity of starchy dishes in the SNAPMe dataset.

### 3.4. Model Behavior: Basis Statement Analysis

The visual estimation prompt elicited interpretable reasoning from all models. Table A2 (Appendix A.3) presents representative basis statements. Opus generated more linguistically detailed descriptions, while Sonnet produced more concise categorizations. Despite Opus’s greater verbal elaboration, quantitative accuracy was not consistently superior, suggesting that linguistic sophistication and numerical estimation represent distinct capabilities in LLMs.

## 4. Discussion

### 4.1. Prompt Design and Model Selection

The reproducibility of nutritional estimation from food images is a challenge even for human experts. Nakayama et al. demonstrated that registered dietitians showed substantial inter-individual variability when estimating nutritional content from food photographs without prior information [12]. Studies of image-based dietary assessment have reported pre-training inter-rater ICC as low as 0.14 among nutritionists [13]. Against this background, the consistent R^2^ values of 0.27–0.60 achieved by Claude Sonnet represent a notable advantage: stable, reproducible estimates without requiring individual calibration. It is also worth contextualizing MedAPE values against traditional dietary assessment methods: 24 h recalls typically show systematic underreporting of 10–30% for total energy, and food frequency questionnaires often show errors exceeding 30–50% for individual nutrients. The MedAPE values observed in this study for macronutrients from packaged food images (14–33% for Sonnet on JBFD) are broadly comparable to or better than traditional self-report methods for food categories where visual information is informative. For meal photographs, MedAPE values of 30–43% for macronutrients are on the higher end but remain within the range of error observed in image-assisted dietary assessment tools. Salt equivalent estimation (MedAPE 34–64%) remains a specific challenge for any visual-only approach, as sodium content is determined by invisible condiments and invisible processing.

The most striking finding is the marked difference between Haiku and Sonnet/Opus. While all models received identical instructions, Sonnet and Opus accurately identified food categories and applied appropriate nutritional knowledge, whereas Haiku produced near-random estimates. Switching Haiku to the visual prompt did not improve performance, confirming that visual recognition capability, not prompt design alone, is the primary determinant of accuracy.

A critical behavioral difference also emerged regarding epistemic honesty. Haiku returned confident numerical estimates regardless of label visibility. Sonnet correctly acknowledged uncertainty by returning null when visual information was insufficient. This difference has direct practical implications: systems built on smaller models may produce confidently wrong estimates, while higher-capability models provide reliable uncertainty signals.

### 4.2. Cost-Performance Tradeoff

Despite Opus costing five times more than Sonnet per API call, MedAPE differences between the two models were consistently small (1–3 percentage points). For JBFD packaged food images, Sonnet actually outperformed Opus for most nutrients. Given that these differences are unlikely to be clinically meaningful, Sonnet offers the superior cost-performance balance for large-scale nutritional estimation applications. An adaptive pipeline, using a lightweight classifier to select between Sonnet for packaged foods and Opus for complex meal photographs, could theoretically optimize both cost and accuracy and represents a promising direction for future work.

### 4.3. Dataset- and Nutrient-Specific Estimation Patterns

**Figure 2:**
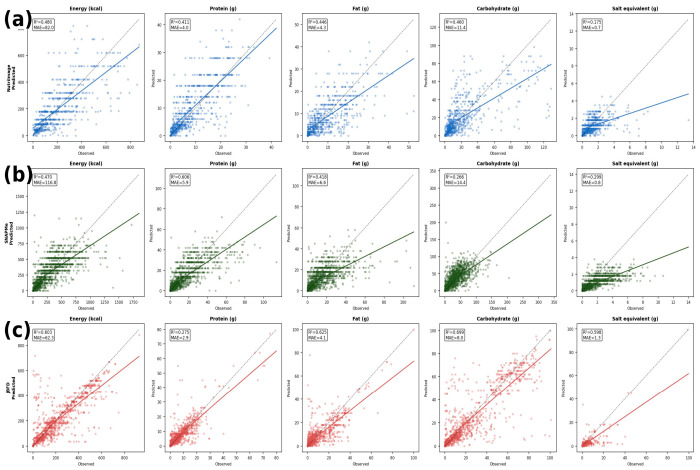
Predicted vs. Observed nutritional values across three datasets (Claude Sonnet 4.6, visual estimation prompt). Dashed line: line of identity. Solid line: linear regression. R^2^ and MAE shown in each panel. (**a**) NutriImage (N = 691); (**b**) SNAPMe (N = 1463); (**c**) JBFD (N = 989). Blue dots: NutriImage; dark green dots: SNAPMe; red dots: JBFD.

The scatter plots in Figure 2 reveal dataset-specific patterns. For JBFD, Sonnet achieved R^2^ of 0.60–0.70 for energy, fat, and carbohydrate, reflecting reliable visual categorization of packaged food products. Protein showed lower R^2^ (0.28), and lipids showed higher variance, as fat content varies substantially within visual categories. Notably, analysis of the predicted values revealed high internal consistency with the Atwater equation [14]: in 79.5% of NutriImage cases, predicted energy was within 10 kcal of the value calculated from predicted macronutrients using the Atwater factors, suggesting that the model internally applies energy–macronutrient consistency constraints. Under this framework, the primary source of estimation error is not nutrient composition per se, but portion size—a limitation that could be reduced by providing reference objects in the image or combining LLM-based composition estimation with a separate portion size estimation model.

For NutriImage, Japanese cuisine presents particular challenges: many dishes involve complex ingredient mixtures (e.g., stewed vegetables, tofu in broth) where macronutrient proportions are not visually apparent. The brownish broth of agedashi tofu and the layered composition of curry rice are examples where visual estimation is inherently ambiguous. That Sonnet achieved R^2^ = 0.480–0.476 under these conditions suggests robust visual recognition capability. Furthermore, the standard Atwater energy conversion factors (4/9/4 kcal/g for protein, fat, and carbohydrate) [14] may fit less well for traditional Japanese cuisine, where substantial energy contributions from invisible ingredients—dashi stock, fermented seasonings, and high-water-content preparations—are difficult to characterize visually, and where high dietary fiber from seaweed and konjac further reduces effective metabolizable energy relative to the Atwater estimate. This structural mismatch between the Atwater framework and Japanese food composition may partly explain the lower R^2^ observed for NutriImage compared with JBFD. Supporting this interpretation, Sonnet achieved comparable energy R^2^ for NutriImage and SNAPMe (0.480 vs. 0.470), but substantially lower protein R^2^ (0.411 vs. 0.606), suggesting that energy-level recognition is dataset-independent, whereas macronutrient allocation—which relies on both visual food categorization and Atwater-consistent distribution—is more sensitive to the opacity of Japanese cuisine composition.

For SNAPMe, real-life mobile photography introduces variability in lighting, angle, and background. The sizing marker included in SNAPMe photographs was not referenced in our prompt; instructing the model to use this marker for portion size estimation may improve accuracy.

Salt equivalent showed consistently poor accuracy (MedAPE 34–64%). Unlike macronutrients, sodium content is determined primarily by invisible condiments added during preparation. Future work should explore hybrid approaches incorporating user-provided annotations to supplement visual estimation.

### 4.4. Comparison with Prior Work

Compared with Rodriguez-Jimenez et al. [6], who used ChatGPT-5 manually on 195 SNAPMe items, our fully automated approach with Sonnet achieved lower energy MAE (−4.9%) and lower MAE for protein (−25.7%) and fat (−24.5%), though carbohydrate MAE was higher (+23.1%). The reference study’s augmented conditions (Cases 2–4) provided additional non-visual information that substantially improved their accuracy. Our image-only condition is more practically deployable, requiring no manual annotation. This comparison underscores that the image-only task faces fundamental limitations that no current approach has overcome, including FoodLog and similar systems.

### 4.5. Data Availability and Future Directions

The consistently poor estimation of salt equivalent suggests that visual information alone is insufficient for sodium estimation. Future work should explore hybrid approaches in which users provide brief structured annotations (cuisine type, cooking method, self-rated saltiness) to supplement visual estimation.

The NutriImage dataset comprises over 880 meal photographs with dietitian-calculated nutritional values, developed in collaboration with a university cooperative cafeteria. Given the research value of this dataset, the authors are currently exploring possibilities for public release. Researchers interested in accessing the dataset prior to public release are encouraged to contact the corresponding author.

### 4.6. Clinical Considerations and Responsible Use

The MedAPE values reported in this study (14–62% across nutrients and datasets) indicate that LLM-based image estimation should not be used as the sole input for dietary management in clinical settings where nutritional precision is critical, such as renal disease (dietary potassium and phosphorus restriction), diabetes (carbohydrate counting), or dyslipidemia (fat intake monitoring). Under- or over-estimation of macronutrients by even moderate margins could compromise therapeutic dietary adherence. Human oversight by a registered dietitian remains essential when these tools are applied in clinical or high-stakes self-monitoring contexts. For population-level epidemiological applications, where group-level trends rather than individual precision are the target, the accuracy observed here may be sufficient. We recommend that developers deploying LLM-based dietary assessment tools clearly communicate estimation uncertainty to end users, specify that outputs are approximate estimates rather than precise measurements, and provide explicit guidance on contexts where professional dietary consultation is required.

### 4.7. Limitations

The present study has several limitations. First, NutriImage used 691 items after quality filtering; SNAPMe and JBFD used all available items (1463 and 989–1000, respectively). Sampling variability was not assessed; repeating the random selection with different seeds would provide more robust performance estimates for JBFD. The current analysis does not report regression calibration (slope and intercept of predicted versus observed values); future work should include calibration metrics to identify systematic directional biases in model estimates. Second, the NutriImage dataset was developed using an earlier edition of the Japanese Standard Tables of Food Composition. Third, the visual estimation prompt was optimized on JBFD images and may not be optimally calibrated for all meal photograph types. Fourth, JBFD images are web-crawled marketing photographs rather than consumer-taken images. Fifth, model versions change rapidly; results may differ with future Claude releases. Sixth, all nutrient estimates are expressed per 100 g edible portion for JBFD and per photographed portion for NutriImage and SNAPMe; translation between these reference units requires additional portion size estimation, which is subject to further error. Seventh, the proportion of null outputs under each prompt condition and how null rates relate to image characteristics (e.g., absence of visible nutrition labels) are not fully characterized in the present study. Eighth, the NutriImage OCR-mask quality audit excluded 21.2% of images; the excluded images may have systematically different visual characteristics.

## 5. Conclusions

We evaluated LLM-based nutritional estimation from food images across three datasets and three model tiers using a visual estimation prompt. Our results demonstrate that: (1) the visual estimation prompt substantially improves accuracy, but only when paired with a sufficiently capable model—the improvement from Haiku default to Sonnet visual reflects both prompt and model effects; (2) Claude Sonnet offers the best cost-performance balance, with MedAPE differences from Opus of only 1–3 percentage points despite a 5-fold cost difference; (3) packaged food images yield higher accuracy than meal photographs for most nutrients; and (4) salt equivalent estimation remains fundamentally difficult. These findings provide actionable guidance for LLM-based dietary assessment system design: for packaged food applications, Sonnet with a visual estimation prompt is recommended; for complex meal photographs, Opus may offer a modest advantage; salt estimation should not rely on visual information alone; and low-capability models are not recommended for automated numerical output. Human oversight and clear communication of estimation uncertainty are essential for responsible deployment.

## Figures and Tables

**Table 1:** Estimation accuracy (R^2^ and MAE) by model and dataset (NutriImage N = 691, SNAPMe N = 1463, JBFD N = 989–1000; visual estimation prompt).

Nutrient	NutriImage						SNAPMe						JBFD					
	Haiku R^2^	Haiku MAE	Sonnet R^2^	Sonnet MAE	Opus R^2^	Opus MAE	Haiku R^2^	Haiku MAE	Sonnet R^2^	Sonnet MAE	Opus R^2^	Opus MAE	Haiku R^2^	Haiku MAE	Sonnet R^2^	Sonnet MAE	Opus R^2^	Opus MAE
Energy (kcal)	0.131	121.1	0.480	82.0	0.506	81.6	0.331	132.3	0.470	116.9	0.530	111.0	0.230	106.3	0.603	62.3	0.586	57.6
Protein (g)	−0.133	6.1	0.411	4.0	0.476	3.8	0.457	7.1	0.606	5.9	0.601	5.7	−0.100	4.6	0.275	2.9	0.236	2.8
Lipids (g)	−0.250	6.6	0.446	4.4	0.409	4.5	0.070	8.0	0.418	6.6	0.464	6.3	0.267	7.9	0.625	4.1	0.681	3.7
Carb (g)	0.209	16.4	0.460	11.4	0.524	11.0	0.254	15.8	0.266	14.4	0.410	13.2	0.152	16.5	0.699	8.1	0.702	7.4
Salt (g)	0.054	0.8	0.175	0.7	0.231	0.7	0.168	0.9	0.299	0.8	0.410	0.7	0.203	1.8	0.598	1.3	0.659	1.1

Gray shading: best performance per dataset per nutrient. Haiku = claude-haiku-4-5-20251001; Sonnet = claude-sonnet-4-6; Opus = claude-opus-4-6. MAE units: kcal for energy, g for all others.

**Table 2:** Median Absolute Percentage Error (MedAPE, %) by model and dataset (NutriImage N = 691, SNAPMe N = 1463, JBFD N = 989–1000; visual estimation prompt).

Nutrient	NutriImage			SNAPMe			JBFD		
	Haiku	Sonnet	Opus	Haiku	Sonnet	Opus	Haiku	Sonnet	Opus
Energy (kcal)	51.5%	35.9%	34.9%	38.6%	33.5%	32.8%	37.7%	19.3%	14.2%
Protein (g)	61.6%	38.8%	38.1%	48.7%	38.5%	38.3%	47.1%	25.0%	21.9%
Lipids (g)	70.1%	52.4%	55.1%	52.2%	46.9%	44.2%	69.8%	39.1%	33.3%
Carb (g)	76.7%	50.4%	46.9%	41.0%	38.1%	35.6%	51.4%	23.4%	19.8%
Salt (g)	49.6%	44.6%	45.4%	63.8%	55.8%	52.2%	54.0%	38.3%	33.9%

MedAPE is preferred over MAPE as it is robust to near-zero true values. Lower values indicate better accuracy. Differences between Sonnet and Opus are consistently 1–3 percentage points across all conditions. Gray shading: best performance per dataset per nutrient. Haiku = claude-haiku-4-5-20251001; Sonnet = claude-sonnet-4-6; Opus = claude-opus-4-6.

**Table 3:** Comparison with Rodriguez-Jimenez et al. [6] on SNAPMe (image-only condition, Sonnet).

Nutrient	ChatGPT-5 MAE (N = 195)	ChatGPT-5 RMSE	This Study MAE (N = 1463)	This Study RMSE	Delta MAE%
Energy (kcal)	123.03	163.80	116.85	184.38	−4.9%
Protein (g)	7.89	11.32	5.86	10.18	−25.7%
Lipids (g)	8.70	12.48	6.57	11.29	−24.5%
Carb (g)	11.72	16.84	14.43	23.21	+23.1%

Delta MAE%: percentage change relative to ChatGPT-5 MAE (negative = improvement). Gray shading: lower MAE than ChatGPT-5 (improvement). Salt equivalent not reported in the reference study.

## Data Availability

All estimation scripts and the visual estimation prompt are publicly available at https://github.com/shnkgw-rincom/food-image-nutrition-llm (accessed on 20 May 2026). The SNAPMe dataset is publicly available from the USDA Ag Data Commons. The NutriImage dataset is available from the corresponding author upon reasonable request. The JBFD nutritional data are available at https://doi.org/10.5281/zenodo.20103327 (accessed on 20 May 2026).

## Appendix A. Prompt Designs and Additional Experiments

### Appendix A.1. Prompt Texts

Default prompt: “Look at this food image and estimate the five mandatory nutritional components per 100g. Respond only in JSON format: {energy_kcal: 0, protein_g: 0, fat_g: 0, carb_g: 0, salt_g: 0}”

Visual estimation prompt (main experiments): “Look at this food image. Estimate the nutritional content based solely on visual appearance—color, shape, ingredients, and portion size. Do NOT read any text, labels, or numbers visible in the image. Use only visual information. Provide a brief one-line explanation of your reasoning. Respond ONLY in JSON format: {energy_kcal: value, protein_g: value, fat_g: value, carb_g: value, salt_g: value, basis: one-line reasoning}”

Chain-of-Thought (CoT) prompt: “Look at this food image. Do NOT read any text or numbers. Step 1: Estimate energy (kcal per 100g) from visual appearance. Step 2: Using that energy estimate and visual characteristics, estimate protein, fat, carbohydrate, and salt equivalent. Respond ONLY in JSON format: {energy_kcal: value, protein_g: value, fat_g: value, carb_g: value, salt_g: value, basis: one-line reasoning}”

### Appendix A.2. Prompt and Model Comparison (JBFD Co-Op Subset, N = 300)

**Table A1:** R^2^ values by prompt condition and model (JBFD Co-op subset, N = 300).

Nutrient	Haiku Default	Haiku Visual	Sonnet Default	Sonnet Visual	Delta
Energy (kcal)	0.235	0.170	—	0.844	+0.609
Protein (g)	−0.109	0.130	—	0.894	+1.003
Lipids (g)	0.286	0.162	—	0.844	+0.558
Carb (g)	0.149	0.070	—	0.842	+0.693
Salt (g)	0.237	0.079	—	0.495	+0.258

Delta: difference between Haiku Default and Sonnet Visual. Dash: Sonnet Default not evaluated at scale (returns null for most items).

### Appendix A.3. Representative Basis Statements by Model

**Table A2:** Representative basis statements generated by each model under the visual estimation prompt.

Food Item	Haiku	Sonnet	Opus
Natto (fermented soybean)	Soy product, soft texture—low fat, moderate protein	Natto package—high protein fermented soy, moderate fat, low salt	Typical pack shape of fermented soybean—protein and fat moderately high
Milk (carton)	White liquid in carton—low-fat dairy equivalent	White liquid in milk carton—typical whole milk composition	Milk carton shape, whitish color—estimated as regular whole milk
Curry rice	Brown sauce with rice—a medium-calorie mixed dish	Curry sauce over white rice—carbohydrate-dominant, moderate fat from roux	Japanese curry rice with dark brown roux—carbohydrate-rich base with moderate fat from sauce

Opus generated more linguistically detailed descriptions than Sonnet, yet quantitative accuracy was not consistently superior, suggesting that verbal elaboration and numerical estimation represent distinct LLM capabilities.

### Appendix A.4. Chain-of-Thought Prompt Experiment (JBFD Co-Op Subset, N = 300)

**Table A3:** Visual estimation prompt vs. Chain-of-Thought prompt (Sonnet, JBFD Co-op subset, N = 300).

Nutrient	Visual R^2^	Visual MAE	CoT R^2^	CoT MAE
Energy (kcal)	0.844	35.97	0.816	37.66
Protein (g)	0.894	1.98	0.877	2.08
Lipids (g)	0.844	2.65	0.838	2.71
Carb (g)	0.842	5.12	0.795	5.55
Salt (g)	0.495	0.76	0.383	0.85

The CoT prompt did not improve accuracy for any nutrient, suggesting that sequential reasoning does not benefit this task when energy estimation itself is uncertain.
